# Pharmacological treatment for pubertal progression in boys with delayed or slow progression of puberty: A small-scale randomized study with testosterone enanthate and testosterone undecanoate treatment

**DOI:** 10.3389/fendo.2023.1158219

**Published:** 2023-04-14

**Authors:** Martin Österbrand, Hans Fors, Ensio Norjavaara

**Affiliations:** ^1^ Department of Paediatrics, Gothenburg Paediatric Growth Research Centre, Institute of Clinical Sciences, Sahlgrenska Academy at University of Gothenburg, Gothenburg, Sweden; ^2^ Endocrine Division, Queen Silvia Children’s Hospital, Gothenburg Department of Paediatrics, Gothenburg, Sweden; ^3^ Näl Uddevalla sjukvården (NU) Hospital Group, Gothenburg, Sweden

**Keywords:** pubertal progression, testosterone, delayed puberty, boys, gonadotropins

## Abstract

**Context:**

The use of testosterone enanthate (TE), 50–75 mg intramuscularly (i.m.)/month, for the treatment of boys with delayed puberty or slow progression to induce puberty is the standard of care (SoC) in Sweden. This treatment is empirical and has not been scientifically evaluated. Replacement therapy in hypogonadal boys/young men in Sweden after induction is mainly performed with testosterone undecanoate (TU), 1,000 mg/3 months. TE is only available on license. TE was deregistered in Sweden in 2006. Therefore, this study was initiated to compare the two products.

**Objective:**

To clinically evaluate pubertal progression with six injections of TE, 75 mg i.m./month (1/3–1/5 of adult dose), compared with two injections of TU, 250 mg i.m./3 months (1/4 of adult dose).

**Trial design:**

In the Pubertal Replacement in Boys Study (PRIBS), boys aged 14–16 years in West Sweden with pubertal delay were randomized in a parallel study to TE or TU for pubertal progression. Inclusion criteria were morning testosterone levels of 0.5–3 nmol/L and testicular volume ≤6 ml. Between June 2014 and Nov 2019, 27 boys were included.

**Methods:**

The primary outcome was testicular enlargement ≥8 ml after 12 months. TU treatment was considered clinically similar if the number of boys with testicular enlargement ≥8 ml was 80%–125% of the number of boys with TE. Fisher’s exact chi-square test was used for this analysis.

**Results:**

Both treatments were well tolerated. Twelve of 14 (86%) TU-treated boys reached the primary outcome and 12/12 in the TE group. Fisher’s exact chi-square testing indicated a one-sided *p*-value of 0.28 (the two-sided *p*-value was 0.483). The TU treatment was considered not clinically different from SoC. A post-hoc study showed 25% power. Therefore, no evidence-based conclusion can be drawn from the results even if the clinical data support a similar effect of the treatments.

**Conclusion:**

The present small-scale study supports that both TE and TU had similar effects in terms of pubertal progression.

**Clinical Trial Registration:**

https://www.clinicaltrials.gov/ct2/home, identifier NCT05417035; https://www.clinicaltrialsregister.eu/ctrsearch/search, identifier EUDRACTEudraCT nr 2012-002337-11.

## Introduction

At puberty, male sexual differentiation restarts with nightly pulsatile stimulating impulses of hypothalamic gonadotropin-releasing hormone (GnRH) to the pituitary gland, which releases luteinizing hormone (LH) and follicle-stimulating hormone (FSH) (1, 2). FSH stimulates spermatogenesis by exerting a trophic effect on Sertoli cells, and LH stimulates the Leydig cells to produce testosterone. This nocturnal increase in gonadotropins results in testis growth. The testicular volume (TV) is 2 ml at the very start of gonadarche, and a TV of 3 ml is considered to mark a transitional phase from prepuberty to puberty (3, 4). A TV of 4 ml is considered the clinical sign of pubertal start, i.e., the gold standard of pubertal onset (5). At a TV of 8 ml or more, growth and masculinization are obvious (6). TV continues to increase for 5 years, eventually reaching adult size. Pubertal delay is usually defined as TV ≤ 3 ml at 14 years of age (5), a definition that does not consider the rate of pubertal progression. Approximately 2.5% (under 2–2.5 standard deviations) of all boys are defined as pubertal delayed.

If puberty does not start at age 14 years, it can be delayed or absent (i.e., hypogonadism). Absent puberty (i.e., hypogonadotropic hypogonadism) is a difficult diagnosis to make unless the patient is 18 years old and has not started puberty. Clinically, patients investigated for delayed puberty can also present slowly progressing puberty with a testis volume of 4–6 ml and early pubertal levels of morning testosterone without increased growth velocity (7). Boys with delayed puberty can have low self-esteem and are often eager to be treated (8).

In Sweden, boys with delayed or slowly progressing puberty are treated (if they want medication) with testosterone enanthate (TE) (Testoviron Depot^®^), 75 mg intramuscularly (i.m.)/month for 6 months. This is approximately 1/3–1/5 of the adult dose used for adult hypogonadism. The boys are called back after 6 months for clinical evaluation and after 12 months to follow up on pubertal progression.

Although we have long clinical experience with TE, it has not been evaluated in clinical trials for children. Although TE treatment is off-label and has not been fully evaluated, it has become the standard of care (SoC) due to the convenience of the injection and because the treatment is considered well-tolerated. Newer testosterone treatment options, such as long-acting testosterone undecanoate (TU) (Nebido^®^), testosterone gels, and testosterone patches have not been evaluated in randomized clinical trials for pubertal induction in boys (9). However, in a recently published retrospective study comparing testosterone gel, TE, and no treatment for 3 months for pubertal induction, all treatments resulted in the progression of puberty (10, 11).

Other treatment options for pubertal development, such as methyl testosterone implants, topical solutions, and oral tablets, are seldom used in Sweden for fear of adverse effects and because of difficulty administering and monitoring the resulting testosterone levels (5, 12).

TE is only available on license in Sweden. TE was deregistered in Sweden in 2006 and is not available on regular drug prescriptions and can only be obtained *via* licensing.

The study was initiated to compare the two products on pubertal progression. Our research hypothesis was that long-acting testosterone administered every 3 months would not be inferior to monthly injections of traditional testosterone for pubertal development. This hypothesis was based on a few observations by the investigators of long-acting TU in doses of 1/4–1/5 of the adult dose, used for pubertal replacement therapy.

## Materials and methods

### Trial design

In this prospective open-label clinical study, the Pubertal Replacement in Boys Study (PRIBS), boys 14–16 years old with delayed puberty in terms of slow pubertal progression were randomized to SoC treatment with TE (Testoviron Depot^®^), 75 mg i.m./month (six injections), or low-dose TU (Nebido^®^), 250 mg i.m./3 months (two injections). Our goal was to conduct a study of pubertal replacement therapy in a way resembling a clinical routine. The subjects were recruited from the Paediatric Growth Research Centre in Gothenburg and the Department of Paediatrics, NU Hospital Group, in Trollhättan, Sweden. The study started in 2014 and was academically sponsored. PRIBS was approved by the regional research ethics committee in Gothenburg (14 September 2012, Dnr 506-12), the European Union Drug Regulating Authorities Clinical Trials Database (EudraCT) (no. 2012-002337-11), and the Swedish Medical Products Agency (Läkemedelsverket) and is registered in Clinical Trials.gov ClinicalTrials.gov NCT05417035. All study personnel were trained in ICH Good Clinical Practice guidelines. Informed consent forms were signed before the study started by the parents, and assent was given by the boys.

The PRIBS patients were seen by one of the three study doctors (the present authors). Three study nurses took all the blood samples for testing and administered the injections. Blood was sampled before 09:00 a.m. All nine PRIBS visits took place at the study sites. The TV was determined using a Prader orchidometer.

The primary objective of this study was to evaluate the effect of TE and TU on pubertal development in boys with delayed puberty or slow pubertal progression. The secondary objectives concerned hormone levels, growth, signs of masculinization, and tolerability.

Since a TV ≥ 8 ml represents mid-puberty (6), it was chosen as the primary outcome 12 months after the start of pubertal induction. A TV ≥ 8 ml is normally reached close to peak height velocity (13). The secondary outcomes were growth for 12 months as well as testosterone, inhibin B, dehydroepiandrosterone sulfate (DHEAS), and gonadotropin levels.

The aim for the TU patients was to have the same morning testosterone levels (4–9 nmol/L) after the first injection as those of boys in transition from early to mid-puberty (4). Safety measures included monitoring blood pressure, pulse, liver enzymes, and hemoglobin (Hb) and registering adverse events.

In Swedish Growth Charts, there are prepubertal growth lines between 11.5 and 15 years. The definition of a start growth spurt in the present study was an increase of 0.3 height SDS (14).

The *inclusion criteria* in PRIBS were as follows:

- signed informed consent;
- two morning testosterone values (07:30–09:00 a.m.) of <3 nmol/L and at the study start of <4 mmol/L;
- TV of 4–6 ml bilaterally (patients with a TV of 2–3 ml could be included if they had pubertal morning testosterone values of 1–3 nmol/L and a pubertal GNRH test); and
- bone age ≥11 years (since there was a change in bone age determination methodology during the study period, this inclusion criterion was not used).
- The *exclusion criteria* in PRIBS were as follows:
- growth spurt;
- untreated hypothyroidism, celiac disease, or steroid medication;
- athletic training >10 h/week; and
- use of anabolic steroids or other drugs.

### Patients and methods

Four patients had a TV of 3 ml but had elevated morning testosterone values (i.e., 1.4, 1.3, 1.8, and 2 nmol/L) and were included. One patient had a TV of 2 ml and a morning testosterone value of 0.6 nmol/L; the GnRH test showed pubertal values.

The boys were examined by study doctors twice before the study, at inclusion, and after 3, 6, and 12 months. The patients were seen by study nurses at inclusion, for injections, and for blood tests on days 0, 2, 7, 30, 60, 90, 180, and 365. Weight, height, and blood pressure measurements were made using the standard instruments at the pediatric clinic. All visits to the clinic were between 07:30 and 09:00 a.m. Data were recorded in case report forms, and an independent monitor from the Sahlgrenska Center for Pediatric Ophthalmology Research at the University of Gothenburg surveilled PRIBS. Adverse events and severe adverse events were recorded.

Boys randomized to TE received six monthly 0.3-ml i.m. injections, each containing 75 mg of TE (250 mg/ml); boys randomized to TU received one 1-ml i.m. injection containing 250 mg of TU (1,000 mg/4 ml) twice, 3 months apart. In PRIBS, the nurses administered the intramuscular testosterone injections, and the medication vials/cartridges were slowly turned up and down 20 times before injection.

LH, FSH, and testosterone levels were evaluated twice before the study start and after 3, 6, and 12 months. Morning testosterone was also evaluated twice before the study start on days 0, 2 and 7 and after 1, 2, 3, 6, and 12 months. Inhibin B was analyzed during the study start and after 12 months. Hb, liver enzymes, pulse, blood pressure, height, and weight were measured before the study start and after 0, 3, 6, and 12 months during the study as a safety measure. Health-related quality of life was assessed during the study start and after 6 and 12 months. Blood samples have been frozen for future analysis of 17β-estradiol (using mass spectrometry), sex hormone-binding globulin (SHBG), and anti-Müllerian hormone (AMH).

### Statistical methods and sample size

The primary outcome was testicular enlargement to ≥8 ml after 12 months; the new treatment would be considered clinically similar if its results (number of patients with testicular size ≥8 ml after 12 months) were 80%–125% of the number of patients with testicular size ≥8 ml after 12 months of traditional treatment.

Since TE is SoC and had not been evaluated for pubertal induction, data were analyzed in agreement with the Swedish Medical Products Agency (Läkemedelsverket). The secondary outcomes were described with mean and SD and were analyzed with an independent-samples t-test.

The sample size was not formally considered in terms of statistical power since TE is SoC and had not been evaluated for pubertal induction; therefore, no power analysis was conducted before this study. Our intention was to recruit 20 patients in the TU group and 20 in the TE group.

#### Interim analysis

The TU dose was based on limited clinical observations of pubertal induction, with doses of 1/4–1/5 of those used for replacement therapy in adult men. An interim analysis of testosterone levels in five patients in the TU group was conducted to explore the possibility of titrating the TU dose up or down for the rest of the study, to avoid overly high or low dosages. The aim of TU treatment was to achieve testosterone levels in the lower range found in mid-puberty (4–9 nmol/L), 2 months after the start of injections (4). If the serum testosterone levels were <4 nmol/L in 3/5 patients, the dose would be increased, and if 2/5 patients had testosterone levels >9 nmol/L, the dose would be lowered.

#### Futility test

So as not to expose the boys to a new treatment that was inferior to SoC, a futility test was performed after 10 patients were included in each group. If the new treatment was statistically inferior or superior, the study would be interrupted. The primary outcome was measured for the analysis, and Fisher’s exact chi-square test was used for this analysis.

#### Bioequivalence and non-inferiority interpretation

The Swedish Medical Products Agency (Läkemedelsverket) requested that a bioequivalence and non-inferiority interpretation of the primary outcome be made after the study; the new treatment would be considered not clinically relevant if the results of TU treatment were outside 80%–125% of the TE results. The statistical analysis of the number of boys was conducted using Fisher’s exact chi-square test.

Statistical analyses were conducted using IBM SPSS version 28 statistical software.

### Laboratory analysis

LH and FSH levels were analyzed at Sahlgrenska University Hospital using a high-resolution chemiluminescence microparticle immunoassay method with a detection threshold of 0.1 mIU/ml. Prepubertal and pubertal testosterone levels were analyzed at the pediatric endocrine laboratory at Gothenburg Eastern Hospital using a sensitive high-resolution modified radioimmunoassay with a sensitivity of 0.03 nmol/L (15).

## Results

### Patient recruitment

**Figure 1:**
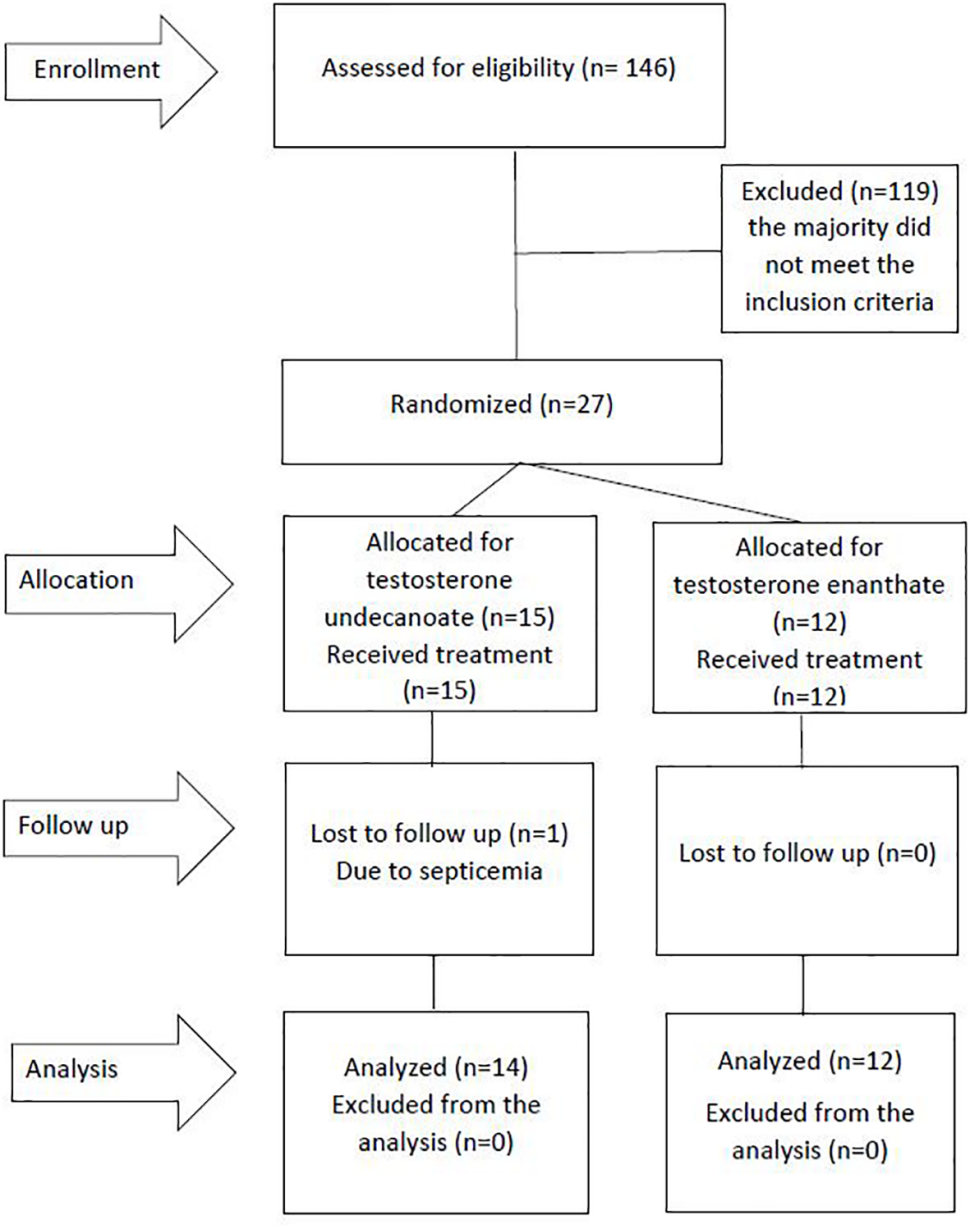
Flowchart of patient recruitment in the Pubertal Replacement in Boys Study.

The first patient was included on 17 June 2014, and the last patient visit was on 7 November 2020. The patient flowchart can be seen in **Figure 1**. The baseline characteristics of the TU and the TE group are visualized in **Table 1**.

**Table 1:** Baseline characteristics of the boys treated with Nebido^®^ and Testoviron Depot®.

Characteristic	Nebido^®^ (TU) n = 15	Testoviron Depot^®^ (TE) n = 12	*p*-Value
Age (year)	14.8 ± 0.5	15.0 ± 0.7	0.40
Height (SDS)	−2.56 ± 0.7	−2.77 ± 0.7	0.51
Height (cm)	152.1 ± 7.4	154.5 ± 6.0	0.37
Weight (kg)	40.8 ± 7.5	47.6 ± 12.2	0.09
Testicular volume right (ml)	4.2 ± 0.9	4.5 ± 1.3	0.48
Testicular volume left (ml)	4.2 ± 0.9	4.6 ± 1.2	0.33
LH (mIE/ml)	1.0 ± 0.6	1.0 ± 0.7	0.99
FSH (mIE/ml)	2.39 ± 2.8	1.9 ± 0.8	0.55
Testosterone (nmol/L)	1.6 ± 0.9	2.7 ± 2.2	0.09
DHEAS	3.2 ± 1.3	3.2 ± 1.37	0.85
Inhibin B (pg/ml)	178 ± 83.3	194 ± 72.9	0.78
Hb (g/L)	132.2 ± 18.5	137.2 ± 10.5	0.41

LH, luteinizing hormone; FSH, follicle-stimulating hormone; DHEAS, dehydroepiandrosterone sulfate; Hb, hemoglobin.

For more details on recruitment, interim analysis, and futility analysis, see **Supplementary Material**.

### Primary outcome

Twelve of 14 boys in the TU group and 12/12 in the TE group reached the primary outcome after 12 months. The boys’ TVs are shown in **Table 2**. The two TU patients who did not reach the primary outcome of a TV ≥ 8 ml started at a TV of 3 ml bilaterally and achieved a TV of 6 ml bilaterally after 12 months. They had a pubertal growth velocity of 8.7–8.9 cm/year, a morning LH value of 3–3.8 mIU/ml at 12 months, and testosterone values of 1.0 and 3.8 nmol/L on day 0 and 6.8 and 10.6 nmol/L at 12 months.

**Table 2:** Testicular volume (TV) and masculinization (PH) at the start and after 6 and 12 months in the Pubertal Replacement in Boys Study in patients treated with Nebido® or Testoviron Depot®.

Treatment	TV start	TV 180 days	TV 365 days	TV ≥ 8 ml	PH start	PH 180 days	PH 365 days
1 Testoviron	4/4	5/6	10/10	Yes	1	3	3
2 Nebido	4/4	5/4	8/8	Yes	2	3	3
3 Nebido	4/4	7/7	12/12	Yes	1	3	4
4 Nebido	5/5	10/10	12/12	Yes	1	4	4
5 Testoviron	6/6	10/12	15/15	Yes	1	4	4
6 Testoviron	5/5	6/6	12/10	Yes	1	2	4
7 Nebido	3/3	6/7	10/10	Yes	1	3	4
8 Nebido	3/3	5/5	6/6	No	1	4	3
9 Nebido	5/5	8/8	15/15	Yes	1	3	4
10 Nebido	5/4	10/12	15/15	Yes	2	3	4
11 Testoviron	3/3	5/6	8/10	Yes	1	2	2
12 Nebido	4/5	8/8	12/12	Yes	2	4	4
13 Nebido	4/4	5/5	8/8	Yes	1	3	3
14 Nebido	5/5	10/8	12/12	Yes	1	4	4
15 Nebido	4/4	8/8	12/12	Yes	1	3	3
16 Testoviron	2/2	6/6	10/10	Yes	1	4	4
17 Nebido	3/3	3/3	6/6	No	1	2	3
18 Testoviron	4/4	5/5	10/10	Yes	1	2	3
19 Testoviron	5/5	8/8	12/12	Yes	1	2	4
20 Nebido	6/6	9/9	12/12	Yes	1	3	4
21 Nebido	4/4	8/8	8/8	Yes	1	2	3
22 Testoviron	3/4	4/5	7/12	Yes	1	3	3
23 Testoviron	5/5	9/9	10/10	Yes	1	3	4
24 Testoviron	5/5	8/8	10/10	Yes	2	4	4
25 Testoviron	6/6	6/6	10/8	Yes	2	3	4
26 Nebido	4/4	6/6	*	*	1	3	*
27 Testoviron	6/6	8/8	10/10	Yes	1	4	4

TV, testicular volume; PH, masculinization.

*Lost to follow-up.

### Bioequivalence and non-inferiority interpretation

The new TU treatment would be considered clinically similar to the TE treatment if it achieved 80%–125% of the TE results. In the TU group, 86% (12/14) of the boys reached the primary outcome versus 12/12 in the TE group. Fisher’s exact chi-square testing indicated a one-sided *p*-value of 0.28 (the two-sided *p*-value is 0.483), so the new treatment was considered not clinically different from SoC.

### Testosterone levels

**Figure 2:**
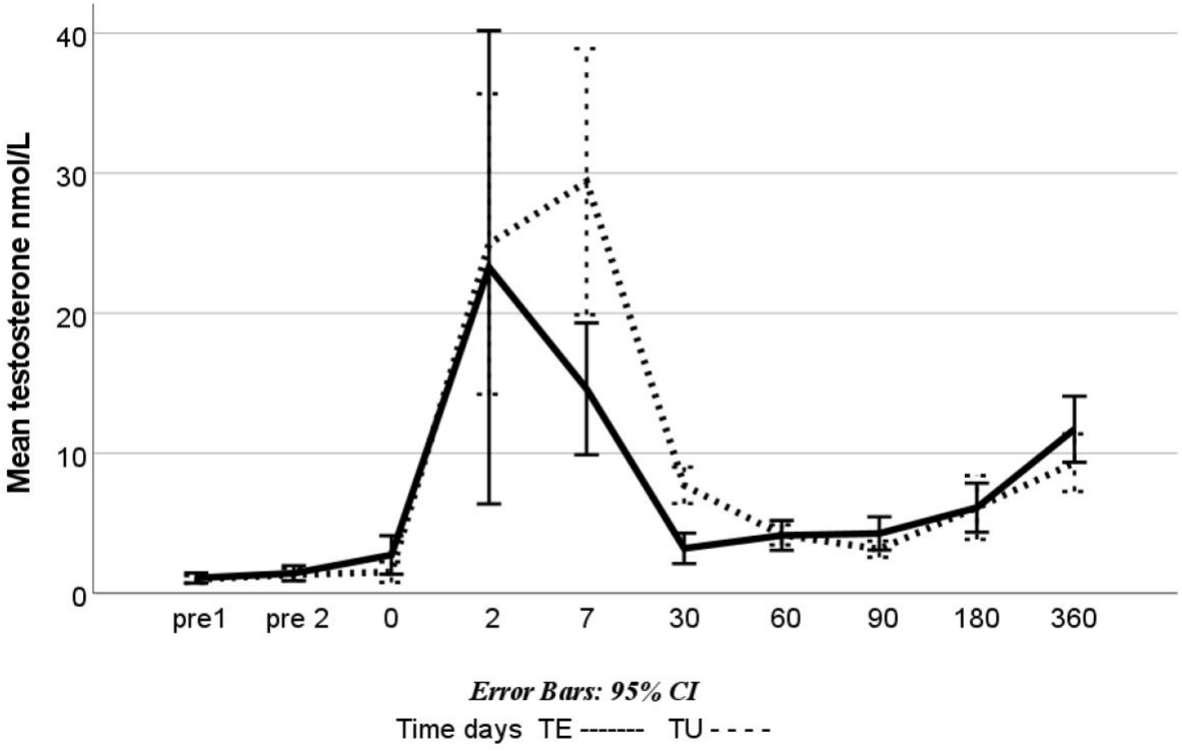
Mean testosterone levels in the Pubertal Replacement in Boys Study: boys treated with Testoviron Depot^®^ (TE) and Nebido^®^ (TU); blood levels of testosterone (nmol/L) were measured between 07:30 and 09:00 a.m. twice before the study start, at the start (0 d), after 2 and 7 days (2 d and 7 d), and after 1, 2, 3, 6, and 12 months (1 m, 2 m, etc.).

Testosterone levels were measured twice before treatment; after 0, 2, and 7 days; and after 1, 2, 3, 6, and 12 months. In both the TU and TE groups, high mean values (i.e., 22.6 and 23.3 mmol/L) were reached 48 h after the first injection. **Figure 2** shows the mean testosterone values in the TU and TE groups for the duration of PRIBS. There was a large variation in testosterone values: 2.9–80 nmol/L in the TE group and 2.7–46.6 nmol/L in the TU group. The mean testosterone levels after 1, 2, and 3 months of treatment in both groups were within our goal interval of 4–9 nmol/L, except in the TU group at 3 months, where it was 3.3 nmol/L, and in the TE group at 1 month, where it was 3.2 nmol/L. **Table 3** shows the minimum, maximum, mean, and standard deviation of testosterone values in the TU and TE groups for the duration of PRIBS.

**Table 3:** Testosterone values in the patients in the Pubertal Replacement in Boys Study.

Testosterone values (nmol/L) in the Testoviron Depot^®^ and Nebido^®^ groups					
		*n*	Minimum	Maximum	Mean/ ± SD
**Nebido^®^**	Pre 1	15	0.1	1.6	0.9 ± 0.5
	Pre 2	15	0.2	2.2	1.3 ± 0.6
	Day 0	15	0.3	3.6	1.6 ± 0.9
	Day 2	14	2.7	46.6	22.6 ± 15.0
	Day 7	12	4.0	46.7	28.4 ± 13.9
	1 month	15	4.2	11.6	7.5 ± 1.8
	2 months	15	2.2	6.3	4.4 ± 1.1
	3 months	15	1.9	5.0	3.3 ± 1.0
	6 months	15	2.9	15.3	6.5 ± 3.0
	12 months	14	3.7	16.6	10.1 ± 3.4
**Testoviron** **Depot^®^**	Pre 1	12	0.5	2.4	1.3 ± 0.6
	Pre 2	12	0.5	3.0	1.4 ± 0.9
	Day 0	12	0.6	3.7	2.7 ± 2.2
	Day 2	12	2.9	80.0	23.3 ± 26.6
	Day 7	12	5.4	30.9	14.6 ± 7.4
	1 month	12	1.3	8.1	3.2 ± 1.7
	2 months	12	1.6	8.1	4.1 ± 1.7
	3 months	12	1.0	7.8	4.3 ± 1.9
	6 months	12	2.3	10.4	6.2 ± 2.8
	12 months	12	6.6	20.7	11.7 ± 3.7

### Gonadotropins

One patient had unmeasurable LH before the study start; 10 patients (8 TE and 2 TU) at 3 months, nine patients (6 TE and 3 TU) at 6 months, and no patient at 12 months had unmeasurable LH. The patient with unmeasurable LH levels at the study start and at 3 and 6 months had an LH level of 2.0 mIU/ml at 12 months and a testosterone level of 0.6 nmol/L at the start, 3.8 at 3 months, 4.3 at 6 months, and 6.8 at 12 months. He was treated with TE and had a TV of 2 ml bilaterally at the start and 10 ml at 12 months. The mean LH levels were similar in the two groups: 1.0 mIU/ml at the start and 2.6 after 12 months in the TU group, versus 1.0 mIU/ml at the start and 2.54 after 12 months in the TE group, with the lowest mean LH values seen at 3 months (i.e., 0.76 and 0.32 mIU/ml in the TU and TE groups, respectively). All patients had measurable FSH values at the start and after 3, 6, and 12 months. Gonadotropin data for the TU and TE groups are presented in **Table 4**.

Table 4Gonadotropin levels in patients in the Pubertal Replacement in Boys Study.

**Table:** 

Gonadotropins (mIE/ml) in the Nebido® group				
	Patients	Minimum	Maximum	Mean ± SD
LH pre1	12	<0.12	2.6	0.9 ± 0.7
LH pre2	13	<0.12	2.2	1.0 ± 0.6
LH 3 months	14	<0.12	3.0	0.8 ± 0.8
LH 6 months	15	<0.12	2.5	1.0 ± 0.8
LH 12 months	14	1.0	6.1	2.6 ± 1.3
FSH pre1	11	0.3	6.8	2.6 ± 2.3
FSH pre2	13	0.2	10.5	2.4 ± 2.8
FSH 3 months	14	<0.12	7.1	2.4 ± 2.2
FSH 6 months	14	<0.12	6.4	2.7 ± 2.2
FSH 12 months	14	0.5	14.4	4.7 ± 4.0

**Table:** 

Gonadotropins (mIE/ml) in the Testoviron Depot® group				
	Patients	Minimum	Maximum	Mean ± SD
	Patients	Minimum	Maximum	Mean/ ± SD
LH pre1	10	<0.12	2.0	0.9 ± 0.6
LH pre2	12	<0.12	2.0	1.0 ± 0.7
LH 3 m	11	<0.12	1.3	0.3 ± 0.4
LH 6 m	12	<0.12	3.3	0.8 ± 1.1
LH 12 months	12	1.4	6.1	2.5 ± 1.5
FSH pre1	11	0.2	3.8	1.9 ± 1.0
FSH pre2	12	0.9	3.6	1.9 ± 0.8
FSH 3 months	11	<0.12	3.2	1.0 ± 1.1
FSH 6 months	12	<0.12	3.8	1.4 ± 1.4
FSH 12 months	12	2.5	7.9	4.0 ± 1.6

LH, luteinizing hormone; FSH, follicle-stimulating hormone.

**Figure 3:**
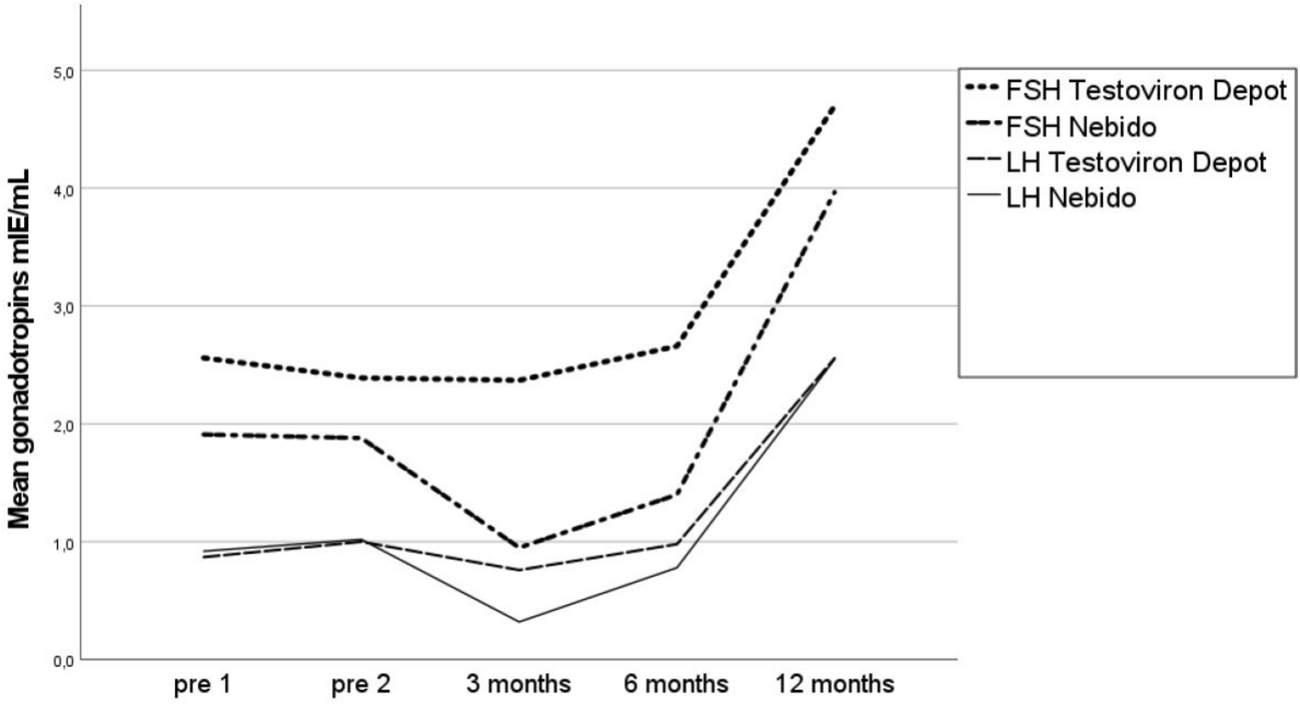
Mean gonadotropins (LH and FSH) in the Pubertal Replacement in Boys Study in the Testoviron Depot^®^ and Nebido^®^ groups measured twice before the study start and after 3, 6, and 12 months. LH, luteinizing hormone; FSH, follicle-stimulating hormone.

The two patients in the TU group who did not reach the primary endpoint had LH values of 1.0 and 2.0 mIE/ml at the study start and of 3.0 and 3.8 mIU/ml at 12 months. Their FSH levels were 5.8 and 10.5 mIU/ml at the study start (the TU group mean was 2.4 mIU/ml) and 14 and 10.7 mIU/ml at 12 months (the TU group mean was 4.8 mIU/ml). The mean LH and FSH levels are shown in **Figure 3**.

For DHEAS and inhibin B, long-term safety, bone age, growth, testicular growth, and signs of masculinization, see **Supplementary Material**.

### Post-hoc analysis

A *post-hoc* power analysis demonstrated 25% power in this study to detect a difference between the groups (16). If this is a true difference (86% *vs.* 100%), we would need 102 patients to have an 80% power to show this difference with an alpha of 0.05.

## Discussion

### Interpretation

There are differences in endogenous testosterone levels after injection with TE versus TU (17). Both treatments produce testosterone peaks in the first week after the injection is given, but with TE, the testosterone level usually remains elevated for less than 1 month, whereas with TU, the level usually remains elevated for 3 months (18).

In PRIBS, a similar testosterone profile was observed in the treated boys, as can be seen in **Figure 2**. Eighty-six percent of the boys in the TU group reached the primary outcome versus 100% in the TE group. In the statistical plan, if the TU group achieved 80%–125% of the TE results, the treatment was considered similar. However, since the post-hoc study power showed 25% (consequently, there is a 75% risk of the results not being correct, no evidence-based conclusion can be drawn from the results, even if the totality of clinical data is supporting a similar effect of the treatments (see below). Nevertheless, the study can be considered a pilot study for future designs of similar studies.

The PRIBS results show that 250 mg of TU administered once every 3 months for 6 months can be used to induce pubertal development in boys with delayed or slow progression of puberty.

Despite the differences in endogenous testosterone levels between the two treatment groups, both treatments resulted in the progression of puberty in all boys. Statistical evaluation of the primary outcome indicated no statistically significant differences between the two treatment groups. An advantage of TU is the feasibility due to longer periods between injections. Of interest is that TE does not seem to suppress the gonadotropins as TU does after 3 and 6 months.

Two TU-treated boys did not reach a testis volume of 8 ml; however, both boys displayed clinical and hormonal signs that puberty had progressed. Both boys had normal LH levels. Their FSH values were 5.8 and 10.5 mIU/ml at the study start and, after 12 months, were the two highest in PRIBS at 10.7 and 14 mIU/ml. They also had the two lowest inhibin B levels at the study start and two of the three lowest inhibin B levels after 12 months. The two boys were followed up after the study. Twelve months after treatment was finished, they had progressed in their pubertal development. Both continued to have a pubertal growth spurt, testicular increase (9–10 ml), and testosterone values of 10.6 and 12.1 nmol/L, which are in the mid-puberty range (4). These results are in line with normal pubertal progression but maybe with a slower pace and support a similar effect of the treatments. A possible explanation is a Sertoli cell dysfunction and a lesser final TV (19).

In both groups, the boys’ puberty had progressed: the mean testis size was 4.1 (TU) and 4.3 ml (TE) at the study start and 9.2 and 10.4 ml at 12 months, and the mean height gain was 9.4 (TE) and 9.2 cm/year (TU). Both groups maintained pubertal levels of endogenous testosterone and gonadotropins with the progression of pubertal growth after treatment stopped. Other studies of the treatment of pubertal delay have shown similarly increased growth rates, i.e., 8.4 and 10.7 cm/year (20, 21).

Whether or not to treat boys (aged 14–16 years) with late or slow progression of puberty is a complicated issue and beyond the scope of this study. Nevertheless, the reason to start treatment is psychological in boys aged 14–16 years (10, 11). From a medical perspective, there is probably no reason to start treatment at that age since puberty will progress in most boys and clinical concerns start at age 16 years (22). In our clinical practice, if a 16-year-old boy presents with delayed or slowly progressing puberty, we recommend inducing pubertal development considering bone health.

### Generalizability

Our clinical centers are the only centers in our region that treat delayed puberty, and all referrals are sent to us, so we conclude that our results are typical of other centers in West Sweden.

Of interest in this context is that in a tertiary center in the UK, 13% of all boys referred for late puberty were treated with testosterone (20), (25). In our study during the observation period, 146 boys were referred for delayed puberty, and 27/146 boys were included (18%). Most boys did not fulfill the inclusion criterion of morning testosterone levels ≤3 nmol/L.

Our findings in PRIBS show that both TU 250 mg/3 months × 2 and TE 75 mg/month × 6 can be used for pubertal progression. They also indirectly suggest that puberty will progress once started, even though the pace might be slow. This is in line with a recent retrospective observational study comparing controls, testosterone gel, and TE where all groups had pubertal progression (11).

### Limitations

Measurements of testis volume (primary outcome) were performed according to clinical practice in pediatric endocrinology by a few (three) doctors. Ultrasound is a more accurate method but is not yet clinically used in our pediatric endocrine unit.

Another limitation of our study is that we lack information about the adult testicle volume from our study cohort.

Although we have long clinical experience with TE, it has not been evaluated in clinical trials for pubertal development. Without these data, it was difficult to power a study to detect a potential difference in new treatments. Therefore, in the present design, the new treatment was considered clinically similar if its results were 80%–125% of the results of traditional treatment.

A limitation is that we do not know the spontaneous progression of puberty in our study cohort. It was not possible to have a control group or a placebo due to the clinical care in our unit. The *post-hoc* power analysis demonstrated *25*% power, and we would have needed 102 patients to have 80% power to show a difference with an alpha of 0.05.

## Data availability statement

The raw data supporting the conclusions of this article will be made available by the authors, without undue reservation.

## Ethics statement

The studies involving human participants were reviewed and approved by The regional research ethics committee in Gothenburg (14 September 2012, Dnr 506-12). Written informed consent to participate in this study was provided by the participants’ legal guardian/next of kin.

## Author contributions

EN designed the study. MÖ wrote the first draft of the manuscript and was responsible for the statistical analysis. MÖ and EN accessed and verified the data. All authors contributed to the last version. All authors contributed to the article and approved the submitted version.
