# Supplementary figures and images for: Pharmacological treatment for pubertal progression in boys with delayed or slow progression of puberty: A small-scale randomized study with testosterone enanthate and testosterone undecanoate treatment
Source: Front Endocrinol (Lausanne). 2023 Apr 14;14:1158219. doi: 10.3389/fendo.2023.1158219 (PMC10140442; doi:10.3389/fendo.2023.1158219)

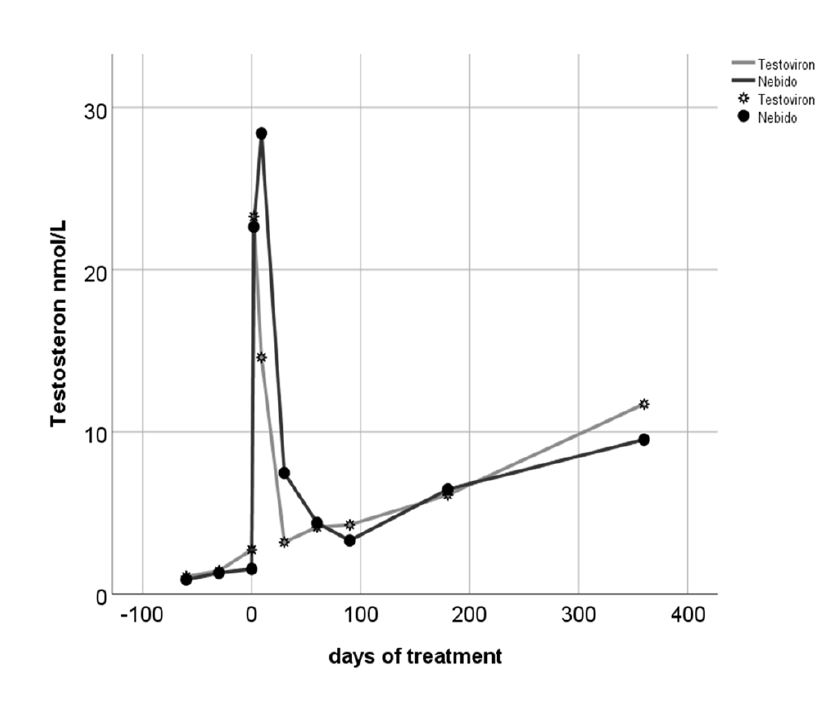

Supplement: Supplementary file 1 [file Image_1.jpeg]
