# Supplementary material for: Pharmacological treatment for pubertal progression in boys with delayed or slow progression of puberty: A small-scale randomized study with testosterone enanthate and testosterone undecanoate treatment
Source: Front Endocrinol (Lausanne). 2023 Apr 14;14:1158219. doi: 10.3389/fendo.2023.1158219 (PMC10140442; doi:10.3389/fendo.2023.1158219)
Supplement: Supplementary file 2 [file DataSheet_1.pdf]

Supplementary Material

Pharmacological treatment for pubertal progression in boys with delayed or slow progression of puberty: A small scale randomized study with testosterone enanthate and testosterone undecanoate treatment.

Martin Österbrand, Hans Fors, and Ensio Norjavaara

Correspondence:

Corresponding author:

Martin. Österbrand martin.osterbrand@vgregion.se

1. Supplementary data

Patient Recruitment

Patient recruitment for PRIBS started on 1 June 2014 and ended on 1 August 2019. Forty boys were recruited based on intention to treat (20 TU and 20 TE). The study was initially approved for three years, by which time only 18 patients had been recruited, so the protocol was amended to allow another two years of recruitment. After five years, 27 patients had been randomized, 12 in the TE group and 15 in the TU group. One patient in the TU group was lost to follow up due to septicemia 11 months after study start.

During this period, 146 boys were referred for delayed puberty. All eligible patients were invited. The majority could not participate because they did not fulfill the inclusion criteria, either having morning testosterone levels >3 nmol/L or growth acceleration. Fear of injections and long distance from the clinic were other reasons for not participating.

Interim Analysis

The aim of TU treatment was to achieve testosterone levels in the lower range found in mid puberty (4–9 nmol/L). Two of five patients in the TU-group had testosterone levels <4 nmol/L and none over 9 nmol/L after 2 months of treatment: their values were 2.4, 2.6, 4.1, 4.5, and 5.8 nmol/L, so the dosage was not changed.

Futility Analysis

After 20 patients were recruited (10 in each group), a futility analysis was conducted. Two of 10 patients (80%) in the TU group did not have a TV ≥8 mL (both patients had a TV of 6 mL) after 12 months. The new treatment was 80% effective, as 8/10 patients reached the primary outcome, whereas 10/10 reached the primary outcome in the traditional treatment group. Fisher's exact chi-square test showed that our hypothesis could not be rejected. A one-sided mid-*p*-value of 0.237 indicates that there is no evidence of a statistically significant difference between the treatment groups.

DHEAS and Inhibin B

The mean DHEAS level for all boys in PRIBS was 3.2 µmol/L (range 1.4–5.6) at study start, 3.14 (1.0–5.8) after 6 months, and 4.2 (1.0–6.9) after 12 months. The mean DHEAS level in the TE group was 3.3 µmol/L at study start and 4.4 after 12 months, while in the TU group it was 3.4 and 4.1 µmol/L at study start and 12 months, respectively.

The Inhibin B range in all boys was 65–341 pg/mL (mean 186 pg/mL) at study start and 62–311 pg/mL (mean 191 pg/mL) after 12 months. At study start, the mean Inhibin B level was 194 pg/mL in

TE-treated patients and 178 pg/mL in TU-treated patients. At 12 months, the mean Inhibin B level was 208 and 193 pg/mL in the TE- and TU-treated patients, respectively. The two boys who did not reach a TV of 8 mL at 12 months had Inhibin B levels of 86 and 65 pg/mL at start and 122 and 62 pg/mL at 12 months. Replicates were analyzed for 10 patients and the CV% varied between 0.6 and 7.1 with a mean of 3.4%.

Long-Term Safety

Blood pressure, pulse, liver enzymes, and Hb were measured four times during the study. Adverse events and severe adverse events were recorded. One patient was lost to follow up due to septicemia 11 months after study start: he had an underlying disorder that predisposed him to septicemia and could not attend the 12-month visit. No other severe adverse events were noted during the study.

Blood pressure was monitored by machine at study start and after 3, 6, and 12 months. Mean BP was 107/65 (52–75/95–129) at study start, 109/61 after 3 months, 110/65 at 6 months, and 111/64 after 12 months (54–74/98–123). All patients had blood pressure in the normal range; no patient developed hypertension. Resting pulse monitoring revealed a mean pulse of 72 at study start, 76 after 3 months, 75 after 6 months, and 71 after 12 months.

The liver enzymes ALAT (normal range 0.36–1.1µkat/L) and ASAT (0,4-0,8) were measured at study start and after 3, 6, and 12 months. No patients had elevated liver enzymes at start. Three patients had transient increases in liver enzymes: one had an elevated ALAT value of 1.6 µkat/L at 3 months, two had values of 1.1 and 1.8 µkat/L at 6 months, and no patients had elevated values at 12 months. Two patients had elevated ASAT values of 1.1 and 2.2 µkat/L at 3 months and three had elevated values of 0.92, 0.99, and 1.4 µkat/L at 6 months; otherwise, the ASAT values were normal. The mean Hb was 135 g/L at study start, 134 after 3 months, 135 after 6 months, and 137 after 12 months; the maximum change in Hb level was a 13 g/L rise.

Bone Age

After PRIBS started, there was a change in methodology from manual to computerized bone age estimation using BoneXpert software, and for the rest of the study bone age was assessed using BoneXpert (with the TW3 model). Bone age was therefore not used as an inclusion criterion, and it was up to the investigator whether or not to consider bone age. 23/27 patients were investigated with Bone Xpert and the mean skeleton age was 11.8 years in the TE group and 11.3 years in the TU group.

Growth

The mean height for the whole group was 153.9 cm before treatment, 156.4 cm after 3 months, 159.4 cm after 6 months, and 163.2 cm after 12 months, for a mean gain of 9.3 cm (range 6.9–12.8). The TU-treated boys had a mean height at study start of 153.4 cm with a height gain of 9.4 cm during the study; in the TE group the mean starting height was 154.5 cm and the height gain was 9.2 cm.

The mean weight for all boys at study start was 43.9 kg (range 32–74 kg). The mean weights for the TE and TU groups were 47.6 and 40.8 kg, respectively, at study start, 50.6 and 43.2 after 3 months, 52.2 and TU 44.9 after 6 months, and 54.6 and 48.4 after 12 months. The mean weight for all boys was 51.3 kg (range 39.5–79.7 kg) after 12 months.

In the TE group the mean height was -2,76 SD at study start and 12 months after treatment start it was -1,97, an increase of 0,78 SD and in the TU treated group the mean was -2,56 at study start and 12 months later -1,96 SD an increase of 0,61 SD

Testicular Growth and Signs of Masculinization

The mean left and right TVs at study start were 4.3 and 4.4 mL (range 2–6 mL). The TE and TU groups had mean TVs of 4.6 and 4.2 mL (range 2–6 mL), respectively, at study start, 7.1 and 7.2 mL (range 3–11 mL) at 6 months, and 10.6 and 10.6 mL (range 6–15 mL) at 12 months.

Tanner staging for pubic hair (PH) was assessed at 0.6 and 12 months. At study start, 5/27 (3 TU and 2 TE) were assessed at PH1 and the rest of the patients were at PH2. After 6 months, five patients were assessed at PH2 (4 TE), 13 at PH3 (5 TE), and 8 at PH4 (4 TE). At 12 months after study start, one patient was assessed at PH2 (1 TE), 9 at PH3 (3 TE), and 16 at PH4 (8 TE).

Testosterone levels

In **figure 2b** the mean testosterone levels for the the TE and TU group are shown at studystart(day0) twice before study start, 2, 7 ,30,60,90,180 and 360 days after studystart.

Legends and Figures

Supplementary Figure 1

Testosterone levels for the TE (Testoviron Depot®) and TU(Nebido®) treated boys. Day 0 is study start.
